# Phloem Proteomics Reveals New Lipid-Binding Proteins with a Putative Role in Lipid-Mediated Signaling

**DOI:** 10.3389/fpls.2016.00563

**Published:** 2016-04-28

**Authors:** Allison M. Barbaglia, Banita Tamot, Veronica Greve, Susanne Hoffmann-Benning

**Affiliations:** Department of Biochemistry and Molecular Biology, Michigan State UniversityEast Lansing, MI, USA

**Keywords:** lipid-binding proteins, phospholipids, lipid signaling, abiotic stress, phloem

## Abstract

Global climate changes inversely affect our ability to grow the food required for an increasing world population. To combat future crop loss due to abiotic stress, we need to understand the signals responsible for changes in plant development and the resulting adaptations, especially the signaling molecules traveling long-distance through the plant phloem. Using a proteomics approach, we had identified several putative lipid-binding proteins in the phloem exudates. Simultaneously, we identified several complex lipids as well as jasmonates. These findings prompted us to propose that phloem (phospho-) lipids could act as long-distance developmental signals in response to abiotic stress, and that they are released, sensed, and moved by phloem lipid-binding proteins (Benning et al., 2012). Indeed, the proteins we identified include lipases that could release a signaling lipid into the phloem, putative receptor components, and proteins that could mediate lipid-movement. To test this possible protein-based lipid-signaling pathway, three of the proteins, which could potentially act in a relay, are characterized here: (I) a putative GDSL-motif lipase (II) a PIG-P-like protein, with a possible receptor-like function; (III) and PLAFP (phloem lipid-associated family protein), a predicted lipid-binding protein of unknown function. Here we show that all three proteins bind lipids, in particular phosphatidic acid (PtdOH), which is known to participate in intracellular stress signaling. Genes encoding these proteins are expressed in the vasculature, a prerequisite for phloem transport. Cellular localization studies show that the proteins are not retained in the endoplasmic reticulum but surround the cell in a spotted pattern that has been previously observed with receptors and plasmodesmatal proteins. Abiotic signals that induce the production of PtdOH also regulate the expression of GDSL-lipase and PLAFP, albeit in opposite patterns. Our findings suggest that while all three proteins are indeed lipid-binding and act in the vasculature possibly in a function related to long-distance signaling, the three proteins do not act in the same but rather in distinct pathways. It also points toward PLAFP as a prime candidate to investigate long-distance lipid signaling in the plant drought response.

## Introduction

As the world population grows, our need for food and fuel increases. This is aggravated by an encroachment of cities on arable land, competition between food and fuel crops, and the impact of global climate changes on crop yields. Abiotic factors such as drought, heat, and cold commonly affect crop yield. To continuously provide sufficient food and fuel for the increasing world population, we need plants with accelerated growth, higher yields, and increased stress tolerance. Since plants are sessile and cannot escape adverse conditions, it is essential to understand how plants perceive environmental changes and how they transmit the signals that convey developmental changes and the resulting adaptations. This requires intracellular and long-distance signaling. The plant long-distance transport systems are the xylem and the phloem. The two main components for phloem transport are sieve elements and companion cells. To enhance transport of molecules through the sieve elements, they optimize the longitudinal flow in these cells by degrading any obstacles in the form of organelles and ribosomes, leaving only the plasma membrane and a thin cytoplasm which contains ER, phloem-specific plastids, and a few dilated mitochondria (van Bel and Knoblauch, 2000; Turgeon and Wolf, 2009). The residual ER is found near the plasmodesmata which connect the sieve elements with the companion cells. It is thought to participate in controlling and mediating the trafficking of proteins and other molecules from the companion cell, where they are synthesized, into the sieve element for long-distance movement (Lucas et al., 2009, 2013). Transport of photoassimilates as well as signaling molecules is thought to occur from source (photosynthetically active, mature leaves) to sink (immature leaves, roots, fruits, flowers, etc.) in a mechanism driven by the osmotic gradient (“Pressure flow hypothesis”; Münch, 1930; for a review see Froelich et al., 2011; Lucas et al., 2013). Our understanding of the phloem has evolved from simple assimilate movement to a complex trafficking system for environmental- and stress signals as well as developmental regulators (Citovsky and Zambryski, 2000; Ding et al., 2003; Wu et al., 2003; Haywood et al., 2005; Lucas et al., 2013) in the form of small molecules (Chen et al., 2001; Corbesier et al., 2003), proteins (Fisher et al., 1992; Schobert et al., 1995; Kühn et al., 1997; Marentes and Grusak, 1998; Kehr et al., 1999; Xoconostle-Cazares et al., 1999; Haebel and Kehr, 2001; Hoffmann-Benning et al., 2002; Giavalisco et al., 2006; Lin et al., 2009; Guelette et al., 2012; Champigny et al., 2013), nucleic acids (Ruiz-Medrano et al., 1999; Citovsky and Zambryski, 2000; Ding et al., 2003; Yoo et al., 2004; Haywood et al., 2005; Pant et al., 2008; Buhtz et al., 2010; Varkonyi-Gesic et al., 2010; Rodriguez-Medina et al., 2011; Hannapel et al., 2013; Pallas and Gómez, 2013), and lipophilic molecules, including complex lipids such as steroids and phospholipids (Madey et al., 2002; Behmer et al., 2011, 2013; Guelette et al., 2012).

Using proteomics approaches, we along with others have identified several putative lipid-binding proteins in the phloem of several plant species as diverse as Perilla, lupine, *Arabidopsis*, broccoli, canola, several cucurbits, poplar, and rice (Table 1; Hoffmann-Benning et al., 2002; Walz et al., 2004; Giavalisco et al., 2006; Aki et al., 2008; Dafoe et al., 2009; Lin et al., 2009; Cho et al., 2010; Rodriguez-Medina et al., 2011; Guelette et al., 2012; Anstead et al., 2013; Lattanzio et al., 2013; Tetyuk et al., 2013; Du et al., 2015).

**Table 1:** **Putative lipid-binding proteins that were identified in the phloem exudates of several plant species (Hoffmann-Benning et al., 2002; Walz et al., 2004; Giavalisco et al., 2006; Aki et al., 2008; Dafoe et al., 2009; Lin et al., 2009; Cho et al., 2010; Rodriguez-Medina et al., 2011; Guelette et al., 2012; Anstead et al., 2013; Lattanzio et al., 2013; Tetyuk et al., 2013; Du et al., 2015)**.

**Protein name/possible function**	**Arabidopsis Accession**	**MW (kDa)**	**Expressed in CCs**	**Lipid ligand**	**Plant Species in which protein was identified**
**LIPID RELEASE/PHLOEM ENTRY**					
Phospholipase Dα2	At1g52570	92	(PLDα1)		Broccoli, poplar
Put. lipase	At4g16820	58			Arabidopsis
GDSL-lipase	At1g29660	40	X	PtdOH, PtdSer	Arabidopsis, poplar, rice, lupine
**CANDIDATES FOR LIPID TRANSPORT/CO-SIGNAL**					
Sec14p-like PtdIns transfer family protein	At1g72160	56	X	Phospholipid-binding pocket	Broccoli
GRP17/oleosin	At5g07530	53	X	PLs	Arabidopsis
Annexin	At1g35720	36	X	PLs	Arabidopsis, pumpkin, rice, canola, castor bean, broccoli, poplar
Flowering locus T	At1g65480	22	X	PtdCho	Arabidopsis, canola, castor bean, cucurbit, rice, lupine
PLAFP1	At4g39730	20	X	PtdOH	Arabidopsis, broccoli
PLAFP2	At1g67280	20			Broccoli
Major latex proteins	At1g24020 At1g70890	18	X		Arabidopsis, rice, lupine
Bet v1 allergen	At1g23130	18	X		Arabidopsis, cucurbits, Rice
Albumin-like protein		16			Cucurbits, Perilla
Acyl carrier proteins	Os11g31900	15			Rice, cucurbits
Dir1/ LTPs	At5g48485	10	X	AzA, LPtdCho	Arabidopsis, cucurbits, rice
**PUTATIVE RECEPTOR COMPONENTS**					
14-3-3 proteins	At1g22300 At2g10450	28; 9	X		Arabidopsis, rice
PIG-P-like protein	At2g39435	50		PtdOH, DAG	
BEACH domain containing protein	At1g03060	400		Contains PH domain; PInsPs?	Broccoli
**OTHER LIPID-INTERACTING PROTEINS WITH POSSIBLE ROLES IN LIPID-MOVEMENT**					
Long-chain-fatty-acid-CoA ligase family protein	At2g04350	68		Lipid metabolism	Broccoli
ARFA1D; phospholipase activator	At1g70490	21	X	Myrosylated; Vesicle formation	Broccoli

*Expression in companion cells is based on Mustroph et al. (2009), Deeken et al. (2008), Zhao et al. (2005); lipid-binding is based on this paper (GDSL; PIG-P), Chen et al. (2008) (ACBP6), Tzen and Huang (1992) (GRP17), Rescher and Gerke (2004) (Annexins), Nakamura et al. (2014) (FT), Benning et al. (2012) (PLAFP); Lascombe et al. (2008); Shah et al. (2014) (DIR1). Proteins examined in this paper are highlighted in yellow*.

The questions arise: What is the function of the phloem lipids? How are they solubilized and transported in this aqueous environment? And what is the role of the phloem lipid-binding proteins in this process?

Our findings led us to propose that phloem (phospho-) lipids could act in long-distance developmental signaling in response to abiotic stress: They could facilitate perception by tethering a signaling molecule, receptor, or secondary messenger to the membrane. Alternatively, they could be (part of) a signal themselves. As such they are released, sensed, and moved by phloem lipid-binding proteins (Benning et al., 2012; Hoffmann-Benning, 2015; Barbaglia and Hoffmann-Benning, 2016). Indeed, the proteins we identified include lipases, that could release the signaling lipid into the phloem, putative receptor components, and proteins that could mediate lipid-movement.

The presence of lipids in an aqueous environment is not without precedence in biological systems: Cholesterol is either taken up into cells and incorporated into membranes, or it is moved to the liver for degradation. Its fate depends on the lipoproteins to which it is bound (for a summary see Nelson et al., 2008). Other examples of the lipid movement and signaling are (I) the developmental regulator Wnt in animals, which requires palmitoleic acid for binding to the receptor Frizzled (Frz; Janda et al., 2012); (II) the platelet activation factor is a phospholipid, which controls platelet aggregation and inflammation (Christie, 2014); (III) the regulation of the β-oxidation by fatty acids via the transcription factor PPARα1 (Wahli and Michalik, 2012). Clearly, lipids can act in long-distance signaling using protein-facilitated mechanisms. The type of protein to which a lipid binds not only determines its transport but also its fate as well as downstream regulatory processes. Despite the fact that these lipid-protein signaling mechanisms are essential for mammalian health and development, their significance in plants is virtually unexplored.

Phloem lipids range from small lipophilic molecules (Jung et al., 2009; Chanda et al., 2011; Chaturvedi et al., 2012; Shah et al., 2014) to lipophilic hormones (Wu et al., 2003; Behmer et al., 2013; Lucas et al., 2013) to (phospho-)glycerolipids (Madey et al., 2002; Guelette et al., 2012; for a summary see Hoffmann-Benning, 2015). Small lipophilic molecules such as oxylipins, dehydroabietinal, a glycerol-3-phosphate-derivative, and azelaic acid (AzA) are studied mostly in the context of biotic stress and systemic acquired resistance (SAR; Howe and Schilmiller, 2002; Chaturvedi and Shah, 2007; Jung et al., 2009; Chanda et al., 2011; Chaturvedi et al., 2012; Shah et al., 2014). The oxylipin jasmonate (JA) is synthesized in response to wounding or herbivory. It moves throughout the plant as its Isoleucine (Ile)- or methyl-ester and elicits a (systemic) defense response (Howe and Schilmiller, 2002; Thorpe et al., 2007; Truman et al., 2007; Mandal et al., 2011; Matsuura et al., 2012; Tamogami et al., 2012). Moreover, a role for the JA-precursor 12-oxo-phytodienoic acid in response to drought and crosstalk with ABA has been suggested (Savchenko et al., 2014). Behmer et al. (2011, 2013) detected free, acylated, and glycosylated derivatives of cholesterol, sitosterol, campesterol, and stigmasterol in the phloem.

*Phospholipids* act as intracellular signals regulating development as well as the response to biotic and abiotic stress (Zhu, 2002; Wang et al., 2007; Munnik and Testerink, 2009; Wang and Chapman, 2012; Gillaspy, 2013; Ischebeck et al., 2013; Hung et al., 2014). One of these, phosphatidic acid is generated in the plasma membrane in response to several environmental stresses and ABA via phospholipases D or C and partakes in intracellular signal transduction (Welti et al., 2002; Wang et al., 2007; Munnik and Testerink, 2009; McLoughlin and Testerink, 2013). However, the concept of phospholipids as long-distance signals has not been investigated and provides a novel aspect in lipid signaling.

To test possible protein-based lipid-signaling pathways, three phloem lipid-binding proteins, which could potentially act in a relay, are characterized here:

- a putative GDSL-motif lipase that may release lipids into the phloem;
- a putative PIG-P protein, with a predicted role in GPI-anchor synthesis and thus, receptor biosynthesis;
- PLAFP (phloem lipid-associated family protein), a putative lipid-binding protein of unknown function.

GDSL esterases/lipases are part of a subfamily of hydrolytic/lipolytic enzymes. They contain a distinct Glycine-Aspartic acid-Serine-Leucine (GDSL) motif and have a flexible active site that changes conformation in the presence of different substrates. This flexible active site leads to a broader substrate- and regiospecificity. It is situated near the N-terminus, while the active site of other lipases is located near the center of the protein (Akoh et al., 2004). GDSL lipases play a role in seed germination (Ling et al., 2006), plant growth and morphogenesis (Brick et al., 1995), and pathogen response (Lee and Cho, 2003; Hong et al., 2008; Oh et al., 2005). *AtGLIP2* plays a role in pathogen defense against *Erwinia carotovora* through the negative regulation of auxin signaling (Lee et al., 2009).

The PIG-P-like protein is a protein of unknown function with similarity to one subunit of the yeast and human phosphatidylinositol N-acetylglucosaminyltransferase subunit P (PIG-P) of the GPI-N-acetylglucosaminyltransferase. The human enzyme transfers N-acetylglucosamine from UDP-N-acetylglucosamine to phosphatidylinositol and assists in the GPI-anchor formation (Watanabe et al., 2000). However, it is much smaller than the putative AtPIG-P, thus their functions are not necessarily related. PIG-P has several homologs in other plants, all containing a DUF4378 at the carboxy-terminus that is shared with the yeast and human PIG-P and could contain the lipid-binding site. The remainder of the plant proteins shows no similarity to any known protein and may have a novel and plant-specific function.

**Figure 1:**
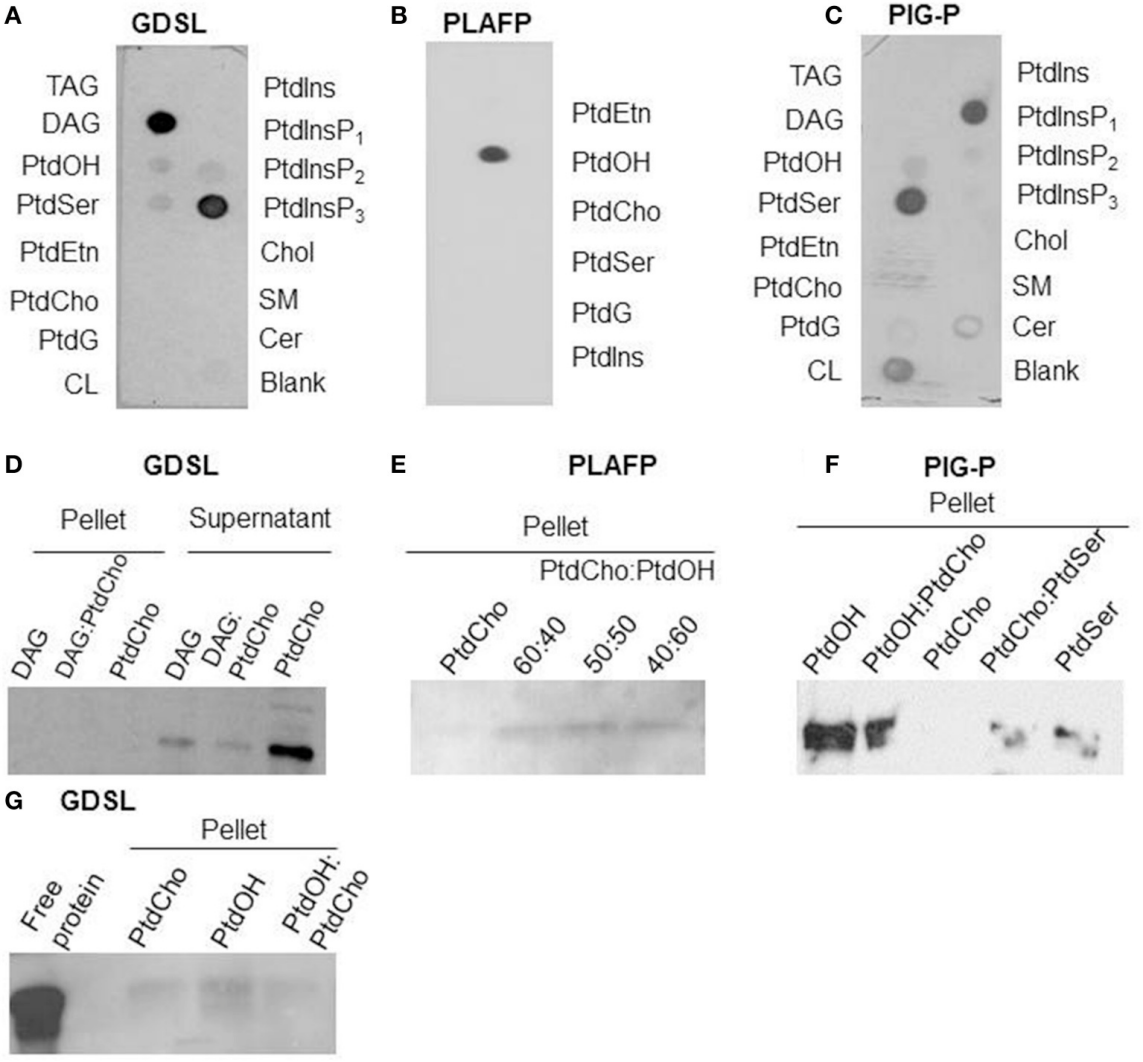
**Lipid-binding properties of the putative GDSL-lipase (A/D/G), the PLAFP (B/E; modified from Benning et al., 2012), and the PIG-P like protein (C/F):** Lipid-binding was examined using protein-lipid overlay assays **(A–C)** and confirmed by liposome-binding assays **(D–G). (D, G)** shows the presence of GDSL-lipase in either pellet or supernatant after incubation with liposomes containing either PtdCho (negative control), DAG, or a mixture. Presence of a band in the pellet indicates binding of the protein to the lipids. E and F show the presence of PLAFP and PIG-P, respectively, in the pellets of liposomes containing PtdOH (PLAFP and PIG-P) and PtdSer (PIG-P) but not if liposomes contain PtdCho alone. TAG, triacylglyceride; DAG, diacylglycerol; PtdOH, phosphatidic acid; PtdSer, phosphatidylserine, PtdEtn, phosphatidylethanolamine; PtdCho, phosphatidyl-choline; PtdG, phosphatidylglycerol, CL, cardiolipin; PtdIns, phosphatidylinositol, PtdInsP_1_, phosphatidylinositol-4-phosphate, PtdInsP_2_, phosphatidylinositol-4,5-phosphate, PtdInsP_3_, phosphatidylinositol-3,4,5- phosphate; Chol, cholesterol; SM, sphingomyelin; Cer, 3-sulfogalactosyl ceramide.

The phloem lipid-associated family protein (PLAFP) is a small putative lipid-binding protein of unknown function. It contains a PLAT/LH2 domain, which is thought to mediate interaction with lipids or membrane-bound proteins (Bateman and Sandford, 1999). Proteins containing the PLAT/LH2 domain are typically stress-induced (Bona et al., 2007; Mhaske et al., 2013). The presence of the PLAT domain has led to the annotation of this protein as a lipase or lipoxygenase, however, PLAFP lacks the catalytic site, suggesting a different function. Hyun et al. (2014) proposed a function in the ER stress response; however, we could not confirm any association with the ER (see Section Localization of Protein and Promoter Activity of GDSL-Lipase, PLAFP, and PIG-P). We have shown that PLAFP specifically binds phosphatidic acid (Benning et al., 2012; see Figure 1C or Figure 1E), a membrane lipid known to participate in intracellular signaling in response to several stresses (Wang, 2005; Wang et al., 2006; Testerink and Munnik, 2011; Arisz et al., 2013).

To participate in any long-distance function, the proteins need to be expressed in the vasculature of the plant. A previous analysis of the phloem translatome by Mustroph et al. (2009) suggested expression of PLAFP and the putative GDSL-motif lipase in phloem companion cells. Our study here goes beyond the proteomics approach that identified putative lipid-binding proteins in the phloem and provides a functional analysis of three candidates in the context of lipid binding and signaling in response to environmental signals. Our findings show that all three proteins are indeed lipid-binding (Figure 1), bind to the same lipid (PtdOH), act in the vasculature (Table 1; **Figure 3**), and respond to PtdOH-mediated stresses (**Figure 4**); However, their different response to environmental factors suggests that the three proteins likely do not act in the same pathway.

## Materials and methods

### Plant growth

*Arabidopsis* seeds were sterilized (20% bleach and 0.5% Triton X-100 for 15 min and washed 6 times with sterile, distilled water) and plated on MS, 1% sucrose, and 0.6% agar. Transgenic lines were selected by growth on plates containing 25 μg/ml kanamycin and confirmed using PCR. For stress experiments plants were germinated on antibiotic-free plates. Next, plates were transferred to 4°C for 2 days before being placed into a Percival growth chamber; 22/18°C, 12-h light/12-h dark photoperiod with 60% relative humidity, and a light intensity of 120 μmol photons m^−2^s^−1^. After 2 weeks seedlings were either transplanted into soil [equal parts Bacto Soil (Michigan Pear Company, Houston), medium vermiculite, and perlite] and grown to maturity or transferred to hydroponic culture for stress experiments.

### Stress treatments

Wildtype Col-0 seedlings were carefully transferred to a hydroponic-like system containing water, covered with a clear plastic dome and left to acclimate for 24 h, at room temperature (22°C.). After this period, 300 mM Mannitol, 150 mM NaCl, 100 μM of ABA, or 30% PEG 6000 were added to the system. Seedlings were harvested at 0, 1, 2, 5, 8, 10, 12, and 24 h post stress (hps). For each time point 3–6 seedlings were pooled. Each time course was performed in triplicate. Asterisks indicate statistical significance as determined by Student's *t*-test; *p* < 0.01.

### Gene expression analyses

Total RNA was extracted from 2–3 week old *Arabidopsis* seedlings or leaves from 5-week- old plants following the instructions provided by the RNEasy Plant Mini Kit (Qiagen). The first strand was synthesized by oligo dT primers using SuperScript First Strand Synthesis III system (Invitrogen). The resultant cDNA was then used for quantitative real-time PCR (qPCR) using SYBR Green (Affymetrix) as the detection probe. Standard conditions (95°C activation, gene-specific annealing temperature, 72°C elongation; repeated 40 times) and a melting curve set at 60°C with a 20 min run time were performed for each run. Primers and annealing temperatures for all the RT-PCR and qPCR are outlined in Supplementary Table 1.

### Protein expression and purification

A cDNA clones for GDSL-lipase (At1g29660), U13183; PLAFP (At4g39730), U21720; and PIG-P (At2g39435) were obtained from *Arabidopsis* Biological Resource Centre, Ohio State University (Columbus, OH, USA). The coding regions of GDSL-lipase, PLAFP, and PIG-P excluding the 78 and 69 nucleotide regions encoding the 26 and 23 amino-acid predicted signal peptides for GDSL-lipase and PLAFP, respectively, was PCR amplified using the primers indicated in Supplementary Table 1, which introduced *Nde*I sites at the 5′ end and BamHI at the 3′ end of the GDSL-lipase and PIG-P PCR products and *Nde*I sites at both ends of the PLAFP PCR product. The PCR products were cloned into pGEMT-Easy vector (Promega), and subcloned into pET15b expression vector (Novagen) using the *Nde*I site to generate the expression clone, pET15b-GDSL-lipase/PLAFP/PIG-P. *E. coli* host strain OrigamiB(DE3)pLysS (Novagen) was transformed with pET15b-PLAFP and BL21(DE3)pLysS for pET15b-GDSL-lipase/ PIG-P, respectively. Transformants were selected by Ampicillin (Amp), Kanamycin (Kan), Chloramphenicol (Cm), and Tetracycline (Tet) resistance for PLAFP and Amp and Cm resistance for GDSL-lipase and PIG-P. IPTG up to the final concentration of 0.5 mM was used to induce protein expression. PLAFP protein was extracted and purified using the HisLink™ resin (Promega) using the HEPES buffers containing different concentrations of imidazole, following the manufacturer's instructions, and GDSL-lipase and PIG-P proteins were extracted and purified using the Ni-NTA resin (Qiagen) using the phosphate buffers containing different concentrations of imidazole, following the manufacturer's instructions. Purification steps include the clear lysate, flow through, wash fraction, and elution fractions. The purified protein was exchanged into 10 mM KH_2_PO_4_ (Lu and Benning, 2009) using a PD10 column (GE healthcare).

### Protein–lipid overlay assay

Ten nmol of various phospholipids (Avanti Polar Lipids; di 18:1 Phosphatidylethanolamine: PtdEtn, Phosphatidic acid: PtdOH, Phosphatidylcholine: PtdCho, Phosphatidylserine: PtdSer, Phosphatidylglycerol: PtdG, Phosphatidylinositol: PtdIns) were spotted onto a Hybond-C membrane (GE Healthcare) for PLAFP-lipid binding studies. Pre-spotted membranes for analysis of PIG-P and GDSL-lipase were purchased from Echelon Biosciences Inc. The protein-lipid overlay assay was performed according to Benning et al. (2012) and Awai et al. (2006).

### Liposome binding assay

Liposomes (lipid-bilayer vesicle) were prepared using the above lipids or a mixture of thereof, following the method described in Awai et al. (2006) and Benning et al. (2012). In short, 250 μg liposomes were mixed with 1 μg of purified protein in 50 mM Tris–HCl, pH7.0, 0.1 M NaCl, and centrifuged at 10,000 × *g* for 10 min at 4°C after incubation at 30°C for 30 min. The pellet was washed and then resuspended in SDS-PAGE sample buffer. Western blot analysis was performed using anti-His and HRP-conjugated goat anti-mouse antibodies.

### GUS reporter gene construct, Arabidopsis transformation, and GUS assay

The 1 Kb region upstream of the transcription initiation site of PLAFP was PCR amplified using the primers indicated in Supplementary Table 1. HindIII and XbaI sites were added at the 5′ and 3′ ends for PLAFP. The PCR product was cloned into pGEMT-Easy vector (Promega) and subcloned into pBI121 (Clontech) vector (from which the 35S promoter was removed by digestion using the restriction enzymes mentioned above) to generate PLAFP1KbPro:GUS which was then transformed into *Agrobacterium tumefaciens* strain GV3101 and C58C1pGV2260 by electroporation, respectively. Positive transformants were selected by Kanamycin resistance, and further confirmed by colony PCR, purified, and sequenced by the Research Technology Support Facility (RTSF) Genomics Core at Michigan State University, and used to transform *Arabidopsis* Col-0 by floral dip method (Clough and Bent, 1998). Transgenic lines were selected by Kanamycin resistance and the incorporation of the transgene was confirmed by PCR, using primers indicated in Supplementary Table 1.

A GUS assay was performed as described (Martí et al., 2010) using GUS staining solution: 50 mM sodium phosphate buffer, pH 7.0, 0.5 mM potassium ferricyanide, 0.5 mM potassium ferrocyanide, 0.1% triton X-100 and 1mg/ml 5-Bromo-4-chloro-3-indoxyl-beta-D-glucuronide cyclohexylammonium salt (Gold Biotechnology). Seedlings were observed under a Nikon Eclipse Ci light microscope.

### Fluorescent reporter gene constructs for GDSL-lipase and PIG-P and PLAFP

The coding sequence of GDSL-lipase, PLAFP, and PIG-P was PCR amplified using the primers indicated in Supplementary Table 1, which added the att sites of the Gateway donor/destination vectors at 5′ and 3′ ends. The PCR product was cloned into pGEMT-Easy vector (Promega) and subjected to the Gateway cloning system where the resultant DNA product was subcloned into the donor vector pDNOR 207 followed by the destination vector, pEarleyGate 103 (or pEarleyGate 102—CFP or pEarleyGate 101—YFP) to generate the clones GDSL1KbPro:GFP (CFP), PLAFP1KbPro:YFP, and PIG-P1KbPro:GFP (CFP), which were then transformed into *A. tumefaciens* strain GV3101 by electroporation. Positive transformants were selected by Kanamycin resistance, further confirmed by colony PCR using the same set of primers mentioned above, sequenced, and used to transiently transform *Nicotiana tabacum*. Leaf samples were then observed under confocal microscopy (Olympus FV1000SP CLSM; YFP Emission wavelength: 530–555 nm, excitation: 515 nm; RFP emission wavelength: 605–630 nm, excitation: 559 nm; CFP emission wavelength: 475–500 nm, excitation: 458 nm) to detect the subcellular localization of the proteins.

## Results

### The predicted phloem lipid-binding proteins GDSL-lipase, PLAFP, and PIG-P-like protein bind lipids

The plant phloem contains several putative lipid-binding proteins (Table 1) as well as lipids (Guelette et al., 2012). To participate in any lipid-based signaling pathway, these proteins need to bind specific lipids, including lipids that can be found in phloem exudates. Protein-lipid overlay assays (Figures 1A–C) suggest a strong interaction of the putative GDSL-lipase with diacylglycerol, phosphatidyl-inositol-3,4,5-trisphosphate (PtdInsP_3_) and a weak interaction with phosphatidic acid (PtdOH); the putative PIG-P shows interaction with phosphatidylserine (PtdSer), phosphatidyl-inositol-4-phosphate (PtdInsP_1_), and PtdOH; PLAFP specifically binds PtdOH.

The lipid-binding seen in the protein-lipid overlay was confirmed using independent liposome-binding assays: the purified putative lipid-binding protein was incubated with liposomes consisting of lipids that had been identified in the overlay assay. Proteins that bind to the liposomes of a specific lipid composition can be detected in the (liposome-containing) pellet (Figures 1D–G, respectively), while proteins that do not bind to the liposomes are found in the supernatant (illustrated in Figure 1D). PtdCho-liposomes were used as negative controls as none of the proteins showed interaction with PtdCho in the protein-lipid overlay. As Figure 1D illustrates, GDSL-lipase is not detected in the pellets containing liposomes that contain DAG or PtdCho, or a mixture thereof. However, it is detected in the supernatant. Different compositions of DAG-containing liposomes were used, none of which interacted with the lipase. On the other hand, the GDSL-lipase does associate with PtdOH-containing liposomes (Figure 1G). Since the binding to PtdOH in the protein-lipid overlay assay was weak an increased amount of protein (10 μg) was used for Figure 1G, showing that in addition to binding PtdOH, there is a weak interaction with PtdCho. Together this indicates that the lipase binds preferentially to PtdOH.

Figure 1E illustrates that PLAFP does not bind to liposomes consisting solely of PtdCho. However, when PtdOH was included in the liposome, PLAFP bound. The amount of protein bound increased with the PtdOH content of the liposomes.

Similarly, PIG-P binds to liposomes containing PtdOH or PtdSer but not to liposomes consisting exclusively of PtdCho (Figure 1F).

These liposome binding studies confirmed binding of PtdOH to PLAFP, PIG-P, and GDSL-lipase as well as binding of PtdSer to the PIG-P-like protein. Binding to PtdInsP_3_ was not tested since this lipid has so far not been reported in plants and is, thus, not of biological relevance (Munnik and Testerink, 2009). Our results demonstrate that all three proteins are indeed lipid-binding proteins. Most importantly, they all bind PtdOH albeit with different intensities. PtdOH is one of the lipids that was found in the phloem (Benning et al., 2012; Guelette et al., 2012) and that is already known to participate in intracellular signaling (Wang et al., 2007; Xue et al., 2009; Hong et al., 2010; Kim et al., 2013; McLoughlin and Testerink, 2013). Thus, these findings suggest the possibility that all three proteins function in a PtdOH-related signaling path.

### Localization of protein and promoter activity of GDSL-lipase, PLAFP, and PIG-P

**Figure 2:**
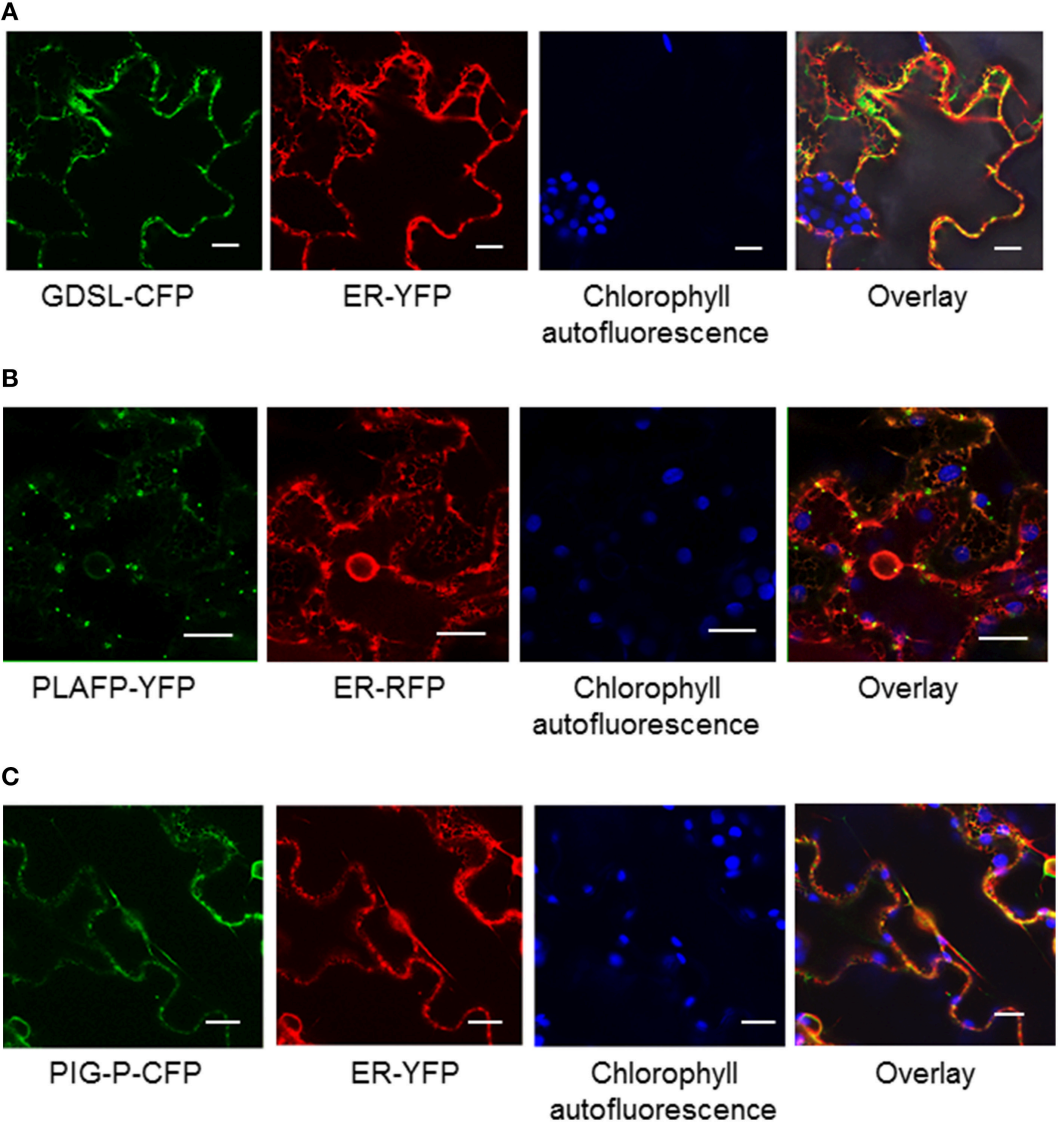
**Localization of GDSL-lipase (A), PLAFP (B), and the PIG-P like protein (C) using C-terminal fluorescent tags and transient expression in tobacco**. Localization of the fusion proteins was determined using confocal microscopy. Chlorophyll fluorescence and a fluorescent ER marker were used as controls. The size marker indicates 20 μm.

During the development of the phloem, many organelles and the nuclei of the sieve elements disintegrate to allow for an unobstructed flow of molecules (Lucas et al., 2013). While some components of the translational apparatus can be found (Lin et al., 2009) they are likely not sufficient for translation and may be remnants of earlier developmental stages. Hence, it is believed, that proteins, RNA, and many other molecules found in the sieve elements are synthesized in the companion cell and move to the sieve elements via plasmodesmata, possibly in an ER-mediated mechanism (Lucas et al., 2013). The GDSL-lipase and PLAFP contain signal peptides, while PIG-P is predicted to be a soluble protein. To understand their localization within the plant cell we generated fusion proteins containing C-terminal fluorescent tags and transiently expressed those in tobacco (Figure 2). All three proteins are localized in a dispersed pattern at the periphery of the cell. No co-localization with chloroplasts or nuclei was observed. Similarly, markers for Golgi and plasma membrane also show no overlap (not shown). Overlays with a fluorescent marker for the ER show that there is little co-localization with the ER marker (Figure 2). This is particularly obvious for GDSL-lipase and PLAFP where cytoplasmic strands containing the ER are clearly visible but show no overlap with the fluorescently tagged protein. PLAFP in particular displays a spotted pattern without ER-colocalization. A similar spotted pattern has been reported for receptors as well as for plasmodesmata-mobile proteins (Kim et al., 2002; Robatzek et al., 2006).

**Figure 3:**
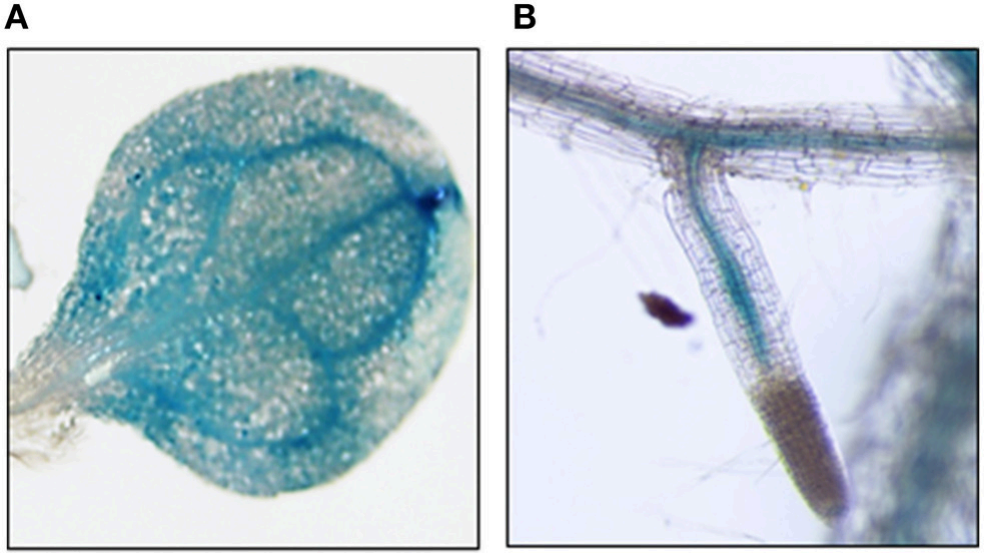
**Promoter Activity via GUS Reporter**. Two-week old *Arabidopsis* seedlings containing the 1 kb region upstream of the transcription initiation site of PLAFP were generated. Gene expression was visualized using a GUS-reporter staining. PLAFP was identified within the leaf vasculature **(A)** as well as the vasculature of root **(B)** of 3 week-old seedlings.

To determine the localization of gene expression we searched several phloem-specific transcriptomes (Zhao et al., 2005; Deeken et al., 2008; Mustroph et al., 2009) for the presence of *GDSL-lipase, PLAFP*, and *PIG-P* gene expression. *GDSL-lipase* and *PLAFP* were found in the companion-cell specific databases suggesting that genes are expressed in the companion cells and could, thus, translocate into the sieve elements via plasmodesmata. Using a GUS reporter gene under the control of the *PLAFP* promoter we show that PLAFP-promoter activity is indeed associated with the vasculature in roots and expanding leaves, as well as in the hydathodes, which are associated with the vasculature (Figure 3: leaf and root). Expression at the branch-point for lateral roots and in the leaf primordia suggests that PLAFP may be necessary during early vasculature development.

Overall gene expression as determined by RT-PCR showed that all three genes are expressed in all tissues of the plant (stem, root, leaf, and flower) with GDSL-lipase and PLAFP expression at slightly reduced levels in the root (Supplementary Figure 1).

### PLAFP and GDSL-lipase gene expression is affected by the same environmental factors that lead to the production of their lipid-ligand phosphatidic acid

**Figure 4:**
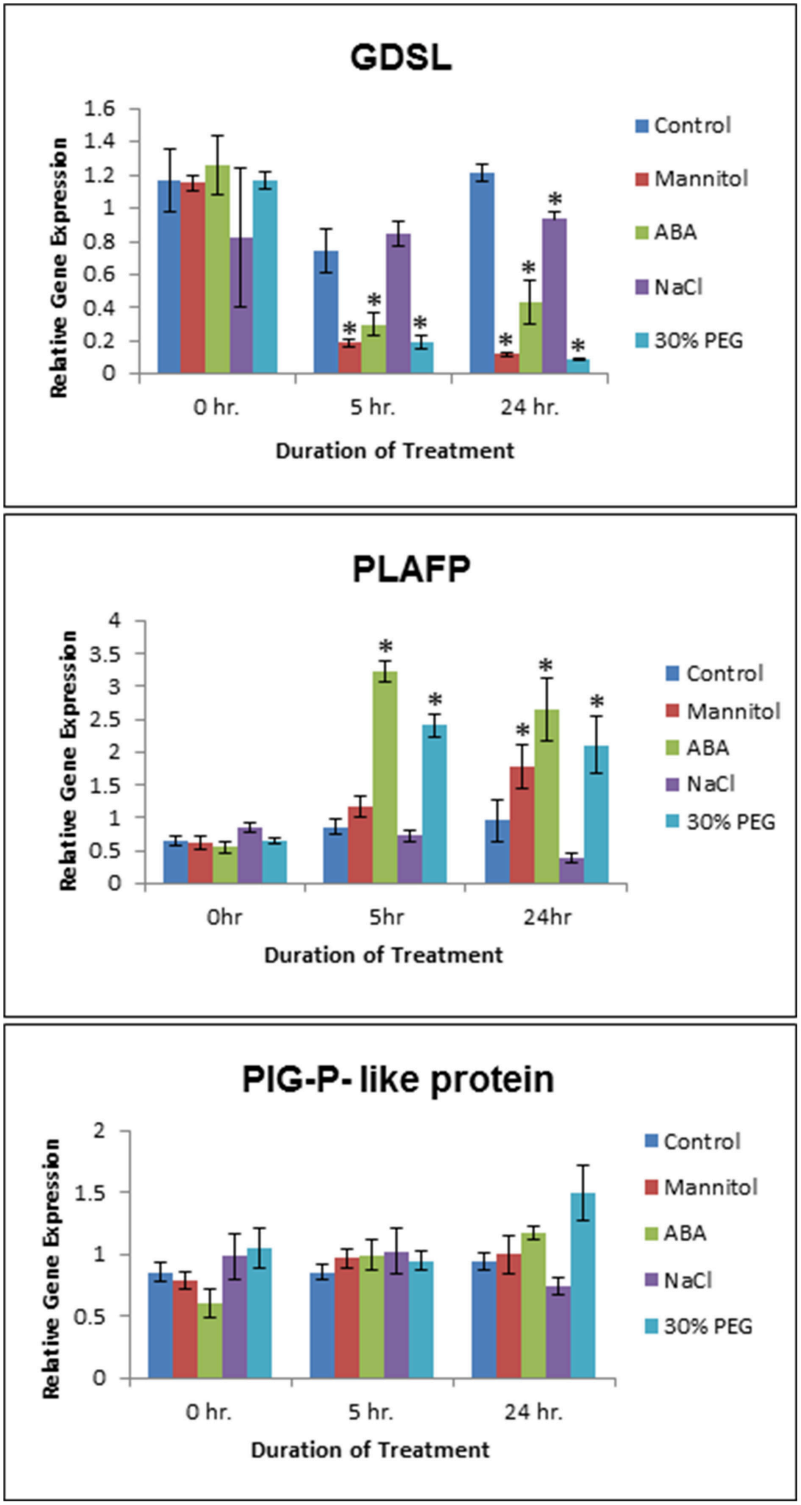
**Effect of Abiotic Stress on ***GDSL***, ***PLAFP***, and ***PIG-P*** expression**. Two week old *Arabidopsis* seedlings were submitted to osmotic (300 mM Mannitol) and salt (150 mM NaCl) stress, a water stress mimic (30% PEG 6000), and ABA (100 μM). Values represent mean and standard error of 3–6 biological replicates as determined using qPCR (three technical replicates per biological replicate). The asterisks indicate significance of *p* < 0.01 (Student's *t*-test).

We have shown that GDSL-lipase, PLAFP, and the PIG-P-like protein bind PtdOH, which we had detected in phloem exudates (Guelette et al., 2012). PtdOH is a well-known intracellular signal acting in response to various abiotic and biotic stresses such as pathogen response and infection, drought, salinity, wounding, cold, cell death, and oxylipin production (Wang et al., 2007, 2014; Munnik and Testerink, 2009; Xue et al., 2009; Hong et al., 2010; Testerink and Munnik, 2011; Kim et al., 2013; Julkowska et al., 2015). It plays a role in maintaining root system architecture as well as salt tolerance (McLoughlin and Testerink, 2013). In addition, PtdOH is widely known for its role in stomatal closure via the activation of the ABA-signaling pathway under drought/osmotic conditions (Guo et al., 2012; Lu et al., 2013; Yao et al., 2013). To understand if GDSL-lipase, PLAFP, and the PIG-P-like protein are controlled by the same environmental factors as PtdOH, we exposed 3-week-old *Arabidopsis* seedlings to salt (NaCl) and osmotic stress (Mannitol) as well as to the drought mimic PEG and the stress signal ABA (Figure 4; Supplementary Figure 2). Concentrations of mannitol, NaCl, PEG, and ABA were based on those used in the literature (Yamaguchi-Shinozaki and Shinozaki, 1994; Nakashima et al., 1997; Zhu, 2002; Fujita et al., 2005; Zhu et al., 2010). Gene expression was monitored for 24 h. *PIG-P* expression was not affected by any of the stress factors (Figure 4). This is not surprising since we had proposed that PIG-P may be part of a receptor and as such, should be constitutively expressed. *GDSL-lipase* expression was downregulated by osmotic (Mannitol) stress, the signaling molecule ABA, and water stress as mimicked by PEG. This downregulation was significant within 5 h of treatment and was maintained for 24 h (Figure 4). PLAFP displayed the opposite response to these stresses: its expression was strongly upregulated by ABA and water stress (PEG) within 2 h, an effect that was maintained for the entire 24-h treatment period (Figure 4). A response to mannitol was observed after 12–24 h. This increase in PLAFP parallels the induction of PtdOH synthesis under the same conditions (Wang et al., 2007, 2014; Munnik and Testerink, 2009; Xue et al., 2009; Hong et al., 2010; Testerink and Munnik, 2011; Kim et al., 2013; Lu et al., 2013; McLoughlin and Testerink, 2013; Yao et al., 2013; Julkowska et al., 2015).

## Discussion

A survey of our proteomics analysis of the phloem exudates of *Arabidopsis thaliana* as well as publications of other phloem proteomes has shown the presence of lipids and lipid-binding proteins within the translocation stream (Table 1; Guelette et al., 2012). This prompted us to propose the possibility of long-distance lipid signaling (Benning et al., 2012). As part of this long-distance path, one protein would release the lipid into the sieve element (GDSL-lipase) where it is bound by a second protein that functions either as transporter or co-signal (PLAFP) and later perceived at a receptor (PIG-P). To participate in the proposed long-distance lipid signaling, these proteins need to fulfill several requirements:

1. They need to bind a specific lipid.
2. Both protein and lipid-ligand need to be present in the phloem sap.
3. Their genes need to be active in the vasculature, specifically in the companion cells. The protein needs to be capable of moving through plasmodesmata into the sieve element.
4. The expression of the gene encoding the lipid-binding protein likely is controlled by the same factors as the production of the lipid-ligand.

Our results show that all three predicted lipid-binding proteins (GDSL-lipase, PLAFP, and the PIG-P–like protein) bind lipids (Figure 1). Most importantly, all three proteins bind PtdOH. This is of particular importance since PtdOH has been found in the same phloem exudates that were used to identify the protein (Guelette et al., 2012). PtdOH is an important intermediate in lipid biosynthesis, a membrane component, and a signaling molecule: As a membrane component it may affect the membrane curvature and, consequently, regulates trafficking and membrane biogenesis (Wang, 2004; Kooijman et al., 2005). Most importantly in the context of this work, PtdOH participates in signaling pathways, often by tethering components of these pathways to the membrane, thus, altering their location and function. PtdOH is rapidly and transiently produced in response to several biotic and abiotic stresses, such as drought, salinity, wounding, cold, pathogen infection, and oxylipin production (Wang et al., 2007, 2014; Munnik and Testerink, 2009; Xue et al., 2009; Hong et al., 2010; Testerink and Munnik, 2011; Kim et al., 2013; Julkowska et al., 2015). The path of its production and the enzymes involved varies depending on the environmental signal (Welti et al., 2002; Uraji et al., 2012; Arisz et al., 2013; McLoughlin and Testerink, 2013; Gonorazky et al., 2014; Julkowska et al., 2015).

We examined if the three PtdOH-binding proteins responded to some of the same environmental factors that induce the production of PtdOH (Figure 4), namely osmotic stress, a water-stress mimic (PEG), and the signaling molecule (ABA). *PIG-P* expression is not influenced by any of those factors. Possible explanations are that PIG-P is part of a receptor and, hence, would likely be constitutively expressed. Alternatively, it could be post-translationally modified or its function could be unrelated to abiotic stress. The expression of *GDSL-lipase* is downregulated by ABA, Mannitol, and PEG and upregulated by NaCl. While there have been other GDSL-lipases that exhibit an increase in expression under various abiotic stresses such as ABA, drought, osmotic, salt, and SA stress (Hong et al., 2008), this particular lipase shows the opposite effect. The most interesting finding was that *PLAFP* expression is upregulated by PEG and ABA within 5 h and by Mannitol within 12 h (Figure 4). Drought, osmotic stress, ABA, salt stress and cold activate distinct phospholipases that cleave phospholipids and generate lipid messengers particularly PtdOH, diacylglycerol, and inositol-3-phosphate. They are thought to affect stress tolerance partially through modulating the expression of stress-responsive genes (Zhu, 2002). One example of a PtdOH-based signaling cascade is the response to osmotic stress and drought, which can lead to an increase in ABA. This increase in ABA leads to the activation of phospholipase Dα1, which in turn produces PtdOH. PtdOH prevents abscisic acid insensitive 1 (ABI1), a protein phosphatase 2C, from binding to the ABA receptor by tethering it to the membrane, subsequently leading to a modification in gene expression and an ABA response. In addition, PtdOH has been shown to be involved in the intracellular signaling process by regulating stomatal closure, which leads to a conservation of water when the plant is experiencing water-deficit conditions (Lu et al., 2013), and by regulating the transcription of genes such as GLABRA2 (GL2) through interaction with the MYB transcription factor, WEREWOLF (Yao et al., 2013). The proposed function here is that PtdOH tethers WEREWOLF to the nuclear envelope and facilitates its movement into the nucleus. In long-distance signaling, PtdOH could either act by binding and moving signals from the companion cell into the sieve element, by tethering a receptor to the plasma membrane of the sieve element, by functioning as binding site for a (proteinaceous) signal, or by being part of a mobile signal that would consist of a mobile protein with a hydrophobic pocket for lipid (PtdOH) binding.

In summary, we find that all three lipid-binding proteins and their lipid ligand PtdOH are present in phloem exudates. GDSL-lipase and PLAFP respond to several abiotic stress factors which also regulate PtdOH-production albeit in opposite fashion. This suggests that these proteins may have a long-distance function in response to abiotic stress. The facts that both, PLAFP and its ligand PtdOH are induced by the same environmental factors, that they are both present in the phloem, and that PLAFP is produced in the vasculature (Figure 3) allow for the possibility that they act in the same signaling pathway and may be part of a mobile signal. Thus, PLAFP-PtdOH can function as model system to study the possibility and mechanisms of lipid-mediated, long-distance signaling in plants. In addition, they provide a unique opportunity as targets for generating stress tolerant plants.

## Author contributions

AB generated GDSL- and PIG-P fluorescently tagged proteins and performed localization studies. She also performed the stress response gene-expression studies and analyzed the GUS expression lines. BT generated the promoter-GUS constructs and the protein constructs for overexpression in *E. coli*. BT, VG, and AB purified the proteins and performed lipid-binding studies. SHB conceived and supervised the experiments. The manuscript was written by SHB with excerpts from AB. BT and VG proofread and approved the manuscript

## Funding

This work was supported by NSF-IOS grant #1144391 to SHB, the USDA-NIFA Hatch project # MICL02233 to SHB, a US Department of Energy graduate assistantship (DE-FG02-91ER20021) and a Cell and Molecular Biology program fellowship to AB, and a MSU-professorial assistantship to VG.

### Conflict of interest statement

The authors declare that the research was conducted in the absence of any commercial or financial relationships that could be construed as a potential conflict of interest.

## Supplementary material

Supplementary Table 1 **Primers and conditions used for Cloning, RT-PCR, and qPCR**.

Supplementary Figure 1 ***GDSL* (A), *PLAFP* (B), and *PIG-P* (C) expression in 5-week old *Arabidopsis* plants**. Values represent mean and standard error of three biological replicates as determined using semiquantitative RT-PCR.

Supplementary Figure 2 **Hydroponic set-up for abiotic stress treatment**. Wildtype seedlings were grown on MS plates for 2 weeks and then transferred to the hydroponic-like set up displayed here. After 24 h of acclimation in their new environment, the abiotic stress treatments were added: osmotic stress received 300 mM Mannitol, salt stress received 150 mM sodium chloride (NaCl), and drought stress signal and mimic in the form of 100 μM abscisic acid (ABA) or 30% polyethylene glycol (PEG), respectively. Seedlings were collected after various time points over a 24 h period. Method adapted from communication with Dr. Patricia Ferreira dos Santos, University of Nevada, Reno.
