# Supplementary material for: Phloem Proteomics Reveals New Lipid-Binding Proteins with a Putative Role in Lipid-Mediated Signaling
Source: Front Plant Sci. 2016 Apr 28;7:563. doi: 10.3389/fpls.2016.00563 (PMC4849433; doi:10.3389/fpls.2016.00563)
Supplement: Supplementary Table 1 — Primers and conditions used for Cloning, RT-PCR, and qPCR. [file Table1.DOCX]

**Supplementary Table 1: Primers used for Cloning, RT-PCR, and qPCR.**

| **Sample** | **Sequence** | **Function, Sites, etc** |
| --- | --- | --- |
| GDSL FW/Rev  At1g29660 | FW: 5’-GCGCAAGCTTGTGTAACAAGACTTAAGGCGC-3’  Rev: 5’-GCGCGGATCCCGATCTCACAAAACAAAACAAAAATAC-3’ | Amplifies ~1000bp upstream of the promoter site; Contains HindIII and BamHI restriction sites; Annealing Temperature: 60°C; Expected Size: 989bp |
| GDSL Clone FW/Rev | FW: 5’-ATGGAGAGTTACTTGAGGAAATGGTG-3’  Rev: 5’-TCAAAGCTGTGCTAATTGCGAGATATC-3’ | Amplifies the entire CDS sequence; Annealing Temperature: 52°C; Expected Size: 1095bp |
| GDSL_GFP FW/Rev | FW: 5'-GGGGACAAGTTTGTACAAAAAAGCAGGCTACCATGGAGAGTTACTTGAGGAAATGGTG-3'  Rev: 5’-GGGACCACTTTGTACAAGAAAGCTGGGTCAAGCTGTGCTAATTGCGAGATATC-3’ | Cloning; Contains att sites; Annealing Temperature: 64°C; Expected Size: 1150bp |
| GDSL F/R | FW: 5’-CATCGATTTCGGCGGCCCCA-3’  Rev: 5’-GTCTTGGCTGCCCTGTGCGA-3’ | RT-PCR; Annealing Temperature: 58°C; Expected Size: 526bp |
| GDSLq FW/Rev | FW: 5’-TCGGCCAACCGAATCTTCAA-3’  Rev: 5’-CCTTCCAATTCCGCAACACG-3’ | qPCR; Annealing Temperature: 52°C; Expected Size: 173bp |
| PLAFP FW/Rev  At4g39730 | FW: 5’-ATGGCTCGTCGCGATGTTCTC-3’  Rev: 5’-AACGACCCAAGAAAGCTTTTTCCG-3’ | Amplifies entire CDS minus the stop codon; Annealing Temperature: 53°C; Expected Size: 543bp |

**Supplementary Table 1 (cont’d)**

| PLAFP-1 F/R | FW: 5’-GAGCAATGGCTCGCTACTGA-3’  Rev: 5’-ACGCCACATTACACTCACAAG-3’ | Amplifies the entire CDS sequence; Annealing Temperature: 52°C; Expected Size: 210bp |
| --- | --- | --- |
| PLAFPq FW/Rev | FW: 5’-TGCTCGACGCAGGATTTTGA-3’  Rev: 5’-CTCAGACCCGACCCGACTAA-3’ | qPCR; Annealing Temperature: 52°C; Expected Size: 126bp |
| pPLAFP FW/Rev | FW: 5’-GCCAAGCTTATTGATTATCATTGCATTGC-3’  Rev: 5’-GCGTCTAGATTTGTTTTTTTCCGGTGAACG-3’ | Amplifies the 1 Kb region upstream of the transcription initiation site of PLAFP; Contains HindIII and Xba restriction sites; Annealing Temperature: 56°C; Expected Size: 1000bp |
| PLAFP-GUS Confirm | FW: 5’-GCCAAGCTTATTGATTATCATTGCATTGC-3’  Rev: 5’-GACCGCATCGAAACGCAGCACG-3’ | Amplifies PLAFP and GUS sequence attached to the C-terminus; used to confirm PLAFP-GUS transgenic lines; Annealing Temperature: 56°C; Expected Size: 1289bp |
| PLAFP-Protein Expression | FW: 5′-GCGCATATGGAAGATGATCCAGACTGTGTATACA-3′  Rev: 5′-GCGCATATGTTAAACGACCCAAGAAAGCTTTTTCCG-3′ | Amplifies the coding region of PLAFP, excluding 69 nucleotide region encoding the 23 amino-acid predicted signal peptide; Contains NdeI restriction site(s); Annealing Temperature: 59°C; Expected Size: 495bp |
| PLAFP_YFP FW/Rev | FW: 5′-GGGGACAAGTTTGTACAAAAAAGCAGGCTACCATGGCTCGTCGCGATGTTCTC-3′  Rev: 5′-GGGGACCACTTTGTACAAGAAAGCTGGGTCAACGACCCAAGAAAGCTTTTTCCG-3′ | Amplifies the coding sequence of PLAFP; contains att sites; Annealing Temperature: 65°C; Expected Size: 601bp |

**Supplementary Table 1 (cont’d)**

| PIG-P FW/Rev  At2g39435 | FW: 5’-GCGCAAGCTTGGGTCAAATTATCTATGTGGTTC-3’  Rev: 5’-GCGCGGATCCTTCTTTTGAGACCTCTACTCTTC-3’ | Amplifies ~1000bp upstream of the promoter; Contains HindIII and BamHI restriction sites; Annealing Temperature: 60°C; Expected Size: 997bp |
| --- | --- | --- |
| PIG-P Clone FW/Rev | FW: 5'- ATGGAGTCTAAGGAAATTAGATCCTCC-3'  Rev: 5'- CTACATGAAGCTGACAACCTCAGAC-3' | Amplifies the entire CDS sequence; Contains att sites; Annealing Temperature: 52°C; Expected Size: 1395bp |
| PIG-P_GFP FW/Rev | FW: 5'-GGGGACAAGTTTGTACAAAAAAGCAGGCTACCATGGAGTCTAAGGAAATTAGATCCTCC-3’  Rev: 5’-GGGGACCACTTTGTACAAGAAAGCTGGGTCCATGAAGCTGACAACCTCAGAC-3’ | Cloning; Contains att sites; Annealing Temperature: 65°C; Expected Size: 1450bp |
| PIG-P F/R | FW: 5’-TTGATGCACTAGCTAAGCCTCCTCA-3’  Rev: 5’-AGCGCAGTGCCTAGCCTCCTA-3’ | RT-PCR; Annealing Temperature: 56°C; Expected Size: 976bp |
| PIG-Pq FW/Rev | FW: 5’-GACGAATTCGGAAGATGCTC-3’  Rev: 5’-TCAGGGTTTCCAGCTGATTC-3’ | qPCR; Annealing Temperature: 50°C; Expected Size: 247bp |
| At18S_F/R | FW: 5’-TCAACTTTCGATGGTAGGATAGTG-3’  Rev: 5’-CCGTGTCAGGATTGGGTAATTT-3’ | Control Primers; Amplifies part of the 18S gene; Annealing Temperature: 50°C; Expected Size: 161bp |
| GUS-F/R | FW: 5’-ATGTTACGTCCTGTAGAAACCCCAACCCGTG-3’  Rev: 5’-AGGAGTTGGCCCCAATCCAGTCCATTAA-3’ | Amplifies GUS gene; Annealing Temperature: 59°C; Expected Size: 976bp |
