# Supplementary material for: Phloem Proteomics Reveals New Lipid-Binding Proteins with a Putative Role in Lipid-Mediated Signaling
Source: Front Plant Sci. 2016 Apr 28;7:563. doi: 10.3389/fpls.2016.00563 (PMC4849433; doi:10.3389/fpls.2016.00563)
Supplement: Supplementary Figure 1 — GDSL (A), PLAFP (B), and PIG-P (C) expression in 5-week old Arabidopsis plants. Values represent mean and standard error of three biological replicates as determined using semiquantitative RT-PCR. [file Presentation1.PPTX]

## Slide 1
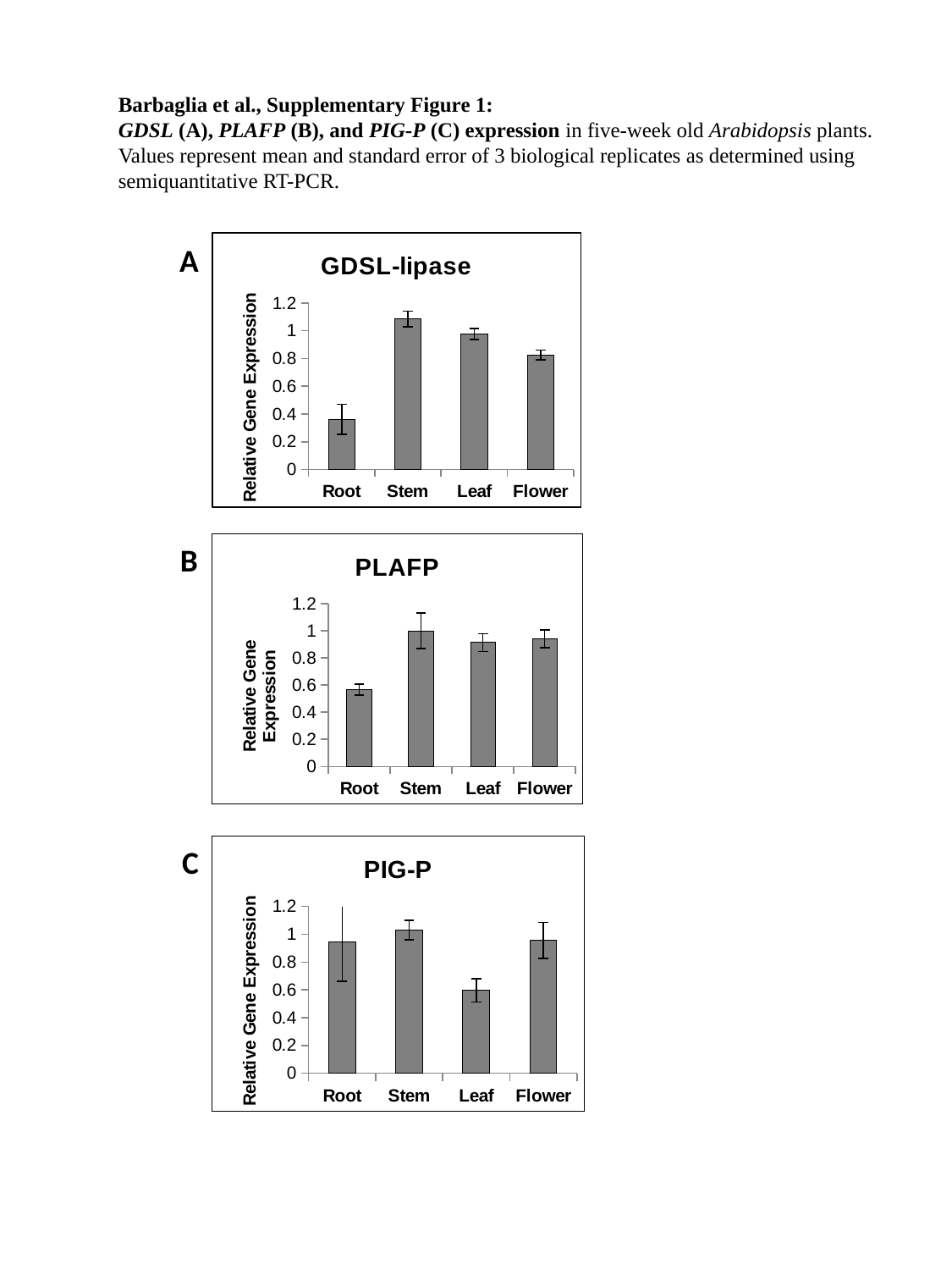

Barbaglia et al., Supplementary Figure 1:
GDSL (A), PLAFP (B), and PIG-P (C) expression in five-week old Arabidopsis plants. Values represent mean and standard error of 3 biological replicates as determined using semiquantitative RT-PCR.
### Chart: GDSL-lipase
| Category | GDSL |
|---|---|
| Root | 0.36107129973061575 |
| Stem | 1.0848474914145607 |
| Leaf | 0.9775560692053912 |
| Flower | 0.8252360974386804 |A
B
### Chart: PLAFP
| Category | |
|---|---|
| Root | 0.5670236000000001 |
| Stem | 0.999856 |
| Leaf | 0.9143420000000001 |
| Flower | 0.940654 |C
### Chart:
| Category | PIG-P |
|---|---|
| Root | 0.9425880094451036 |
| Stem | 1.030053439842131 |
| Leaf | 0.5962704827601555 |
| Flower | 0.9561971991039178 |
