# Supplementary material for: Phloem Proteomics Reveals New Lipid-Binding Proteins with a Putative Role in Lipid-Mediated Signaling
Source: Front Plant Sci. 2016 Apr 28;7:563. doi: 10.3389/fpls.2016.00563 (PMC4849433; doi:10.3389/fpls.2016.00563)
Supplement: Supplementary Figure 2 — Hydroponic set-up for abiotic stress treatment. Wildtype seedlings were grown on MS plates for 2 weeks and then transferred to the hydroponic-like set up displayed here. After 24 h of acclimation in their new environment, the abiotic stress treatments were added: osmotic stress received 300 mM Mannitol, salt stress received 150 mM sodium chloride (NaCl), and drought stress signal and mimic in the form of 100 μM abscisic acid (ABA) or 30% polyethylene glycol (PEG), respectively. Seedlings were collected after various time points over a 24 h period. Method adapted from communication with Dr. Patricia Ferreira dos Santos, University of Nevada, Reno. [file Presentation2.PPTX]

## Slide 1
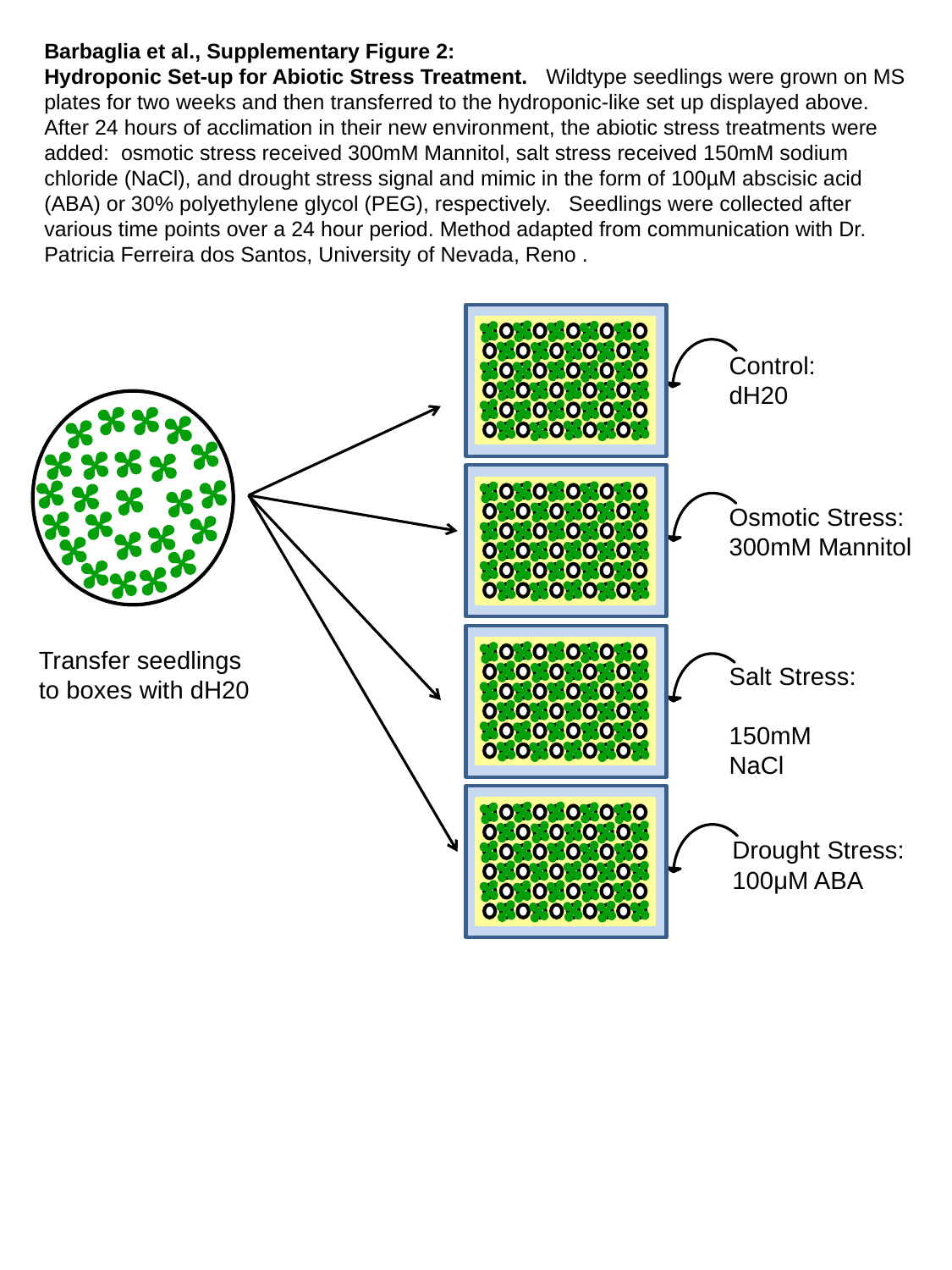

Barbaglia et al., Supplementary Figure 2:
Hydroponic Set-up for Abiotic Stress Treatment. Wildtype seedlings were grown on MS plates for two weeks and then transferred to the hydroponic-like set up displayed above. After 24 hours of acclimation in their new environment, the abiotic stress treatments were added: osmotic stress received 300mM Mannitol, salt stress received 150mM sodium chloride (NaCl), and drought stress signal and mimic in the form of 100µM abscisic acid (ABA) or 30% polyethylene glycol (PEG), respectively. Seedlings were collected after various time points over a 24 hour period. Method adapted from communication with Dr. Patricia Ferreira dos Santos, University of Nevada, Reno .
Control:
dH20
Osmotic Stress:
300mM Mannitol
Transfer seedlings to boxes with dH20
Salt Stress:
150mM NaCl
Drought Stress:
100μM ABA
